# Post-natal steroid exposure in very low birthweight neonates and associations with acute kidney injury

**DOI:** 10.1038/s41372-024-02011-4

**Published:** 2024-05-23

**Authors:** Cassandra Coleman, Jeffrey King, David T. Selewski, Jill C. Newman, Heidi J. Steflik

**Affiliations:** 1 Department of Pediatrics, Vanderbilt University Medical Center, Nashville, TN USA; 2 Department of Pediatrics, Medical University of South Carolina, Charleston, SC USA

**Keywords:** Acute kidney injury, Endocrine system and metabolic diseases, Risk factors, Drug therapy, Paediatrics

## Abstract

**Objective:**

The relationship between adrenal insufficiency (AI), post-natal steroids (PNS) and neonatal acute kidney injury (AKI) remains understudied. We investigated associations between PNS and AKI in very low birthweight (VLBW) neonates, hypothesizing PNS is associated with reduced AKI.

**Study design:**

We conducted a single-center retrospective review of VLBW infants comparing those with and without PNS exposure. Associations between PNS exposure and AKI were evaluated using generalized linear mixed-modeling adjusted for confounders.

**Result:**

Of 567 neonates, 97 (17.1%) were exposed to PNS and 130 (22.9%) experienced AKI. Infants with PNS had lower gestational age, birthweight, Apgar scores, and experienced more AI versus those without PNS (all *p* < 0.05). PNS was associated with AKI (aRR 1.72, 95% CI 1.09–2.72) though hydrocortisone alone was not.

**Conclusion:**

PNS exposure, but not hydrocortisone alone, is associated with increased AKI in VLBW neonates. Further analysis is needed to investigate the role of AI and AKI.

## Introduction

Acute kidney injury (AKI) occurs commonly in the neonatal intensive care unit (NICU) and is associated with increased morbidity and mortality. In the Assessment of Worldwide Acute Kidney Injury Epidemiology in Neonates (AWAKEN) study, Jetton et al. found that AKI in neonates was associated with both increased mortality as well as increased length of stay (LOS) [1]. Furthermore, survivors of neonatal AKI are at increased risk for the development of chronic kidney disease (CKD) [2].

Extremely premature and very low birthweight (VLBW, i.e., birthweight <1500 g) infants are particularly susceptible to AKI. With up to 60% of nephrogenesis occurring in the third trimester, premature birth alters final nephron number significantly, increasing risk of AKI in this population [3, 4]. In the AWAKEN study, incidence of AKI in infants born at less than 29 weeks of gestation was nearly 48% [1]. Other authors have similarly found that incidence of AKI differs significantly by gestational age (GA), with AKI rates being significantly higher with decreased GA and birthweight (BW) [5]. AKI has also been found to be associated with extrarenal outcomes including intraventricular hemorrhage (IVH) [6–8], increased length of mechanical ventilation and bronchopulmonary dysplasia (BPD) in VLBW neonates [9, 10].

Corticosteroids are utilized in VLBW infants for pressor-resistant hypotension, adrenal insufficiency (AI), pulmonary inflammation, air way edema in preparation for extubation, and systemic inflammatory response-type syndromes. Small single-center investigations have suggested that some AKI in VLBW neonates may be due to glucocorticoid-responsive circulatory collapse and relative innate AI, with resolution of AKI upon initiation of steroids, particularly hydrocortisone [11–14]. To our knowledge, this association between corticosteroids and AKI has not been investigated in a large cohort of VLBW neonates.

To address this gap in knowledge, we performed a detailed evaluation of post-natal steroid (PNS) exposure and AKI in VLBW neonates. The objectives of this study were to describe the incidence of AKI in VLBW infants stratified by PNS receipt and investigate potential associations between PNS treatment and AKI with a specific evaluation examining associations between hydrocortisone and AKI. We hypothesized that in VLBW infants, PNS would be associated with reduced incidence of AKI.

## Materials/subjects and methods

### Study population

We conducted a retrospective chart review of VLBW infants born between January 1, 2018, and December 31, 2020, admitted to the Medical University of South Carolina (MUSC) NICU within 48 h of delivery. Infants were identified using the MUSC Internal Perinatal Information System database. We excluded infants who: died or were transitioned to palliative care courses within 48 h of admission, had lethal chromosomal anomalies, had known congenital kidney or urinary tract abnormalities, or had less than two measured serum creatinine values. This protocol was approved by the MUSC Institutional Review Board with a waiver of informed consent.

### Data collection

Demographic information collected included GA, BW, Apgar scores, size for GA (small for gestational age (SGA), defined as <10th percentile for age and large for gestational age (LGA), defined as >90th percentile [15]), sex, race, and AI (defined as presence of diagnosis in discharge diagnoses). Potential confounding factors for AKI that were collected included hypotension, caffeine exposure, patent ductus arteriosus (PDA), sepsis, mechanical ventilation, necrotizing enterocolitis, and nephrotoxic medication exposure [16]. For patients without AKI, we investigated these confounders if present at any time during their hospital stay. For patients with AKI, we screened for these confounders both 48 h before diagnosis of AKI as well as throughout the AKI episode.

Study data were collected and managed using REDCap electronic data capture tools hosted at MUSC [17, 18].

### Exposure: post-natal corticosteroids

The exposure of interest was PNS receipt. If AKI occurred, PNS was considered present only if it was prescribed prior to the first AKI episode. PNS included systemic dexamethasone, prednisone, hydrocortisone, methylprednisolone, or fludrocortisone. Antenatal steroid exposure was excluded as nearly all included infants received antenatal steroids prior to delivery.

### Outcome: neonatal acute kidney injury

Our outcome of interest was neonatal AKI, diagnosed and staged based on severity using the modified, neonatal Kidney Disease: Improving Global Outcomes (KDIGO) serum creatinine criteria [19] (Supplementary Table 1). Only information regarding the first episode of AKI was included. Recurrent episodes of AKI were not evaluated. Notably, AKI diagnosis on the discharge summary, indicating AKI was detected by the neonatal care team during the hospitalization, was also collected, however our analysis and AKI stratification was based on presence or absence of AKI as determined by the research team.

### Statistical analysis

Comparisons were made between those with and without PNS exposure as well as with and without AKI using chi-square, Student’s t, and Wilcoxon rank sum tests as appropriate. Categorical data are expressed as frequency (proportions). Continuous data are expressed as median [interquartile range] for non-normally distributed data and mean ± standard deviation for normally distributed data.

Associations between PNS exposure and AKI were evaluated using bivariate and multivariate generalized linear mixed modeling (GLMM), adjusted for significant confounders for AKI. Outcomes were presented as unadjusted and adjusted relative risks with 95% confidence interval (CI). In multivariable analysis, potential confounders were entered into the model as a covariate if they were statistically significant in the baseline demographic and characteristic analysis (*p* < 0.05) or were established confounders for AKI and found to be associated with AKI in our cohort in bivariate analysis (*p* < 0.05). Adjusted models included PNS, SGA, BW, APGAR scores (1 min), AI, caffeine exposure, hypotension, PDA, sepsis, mechanical ventilation, necrotizing enterocolitis, and nephrotoxic medication exposure. A secondary, subgroup analysis between solely HC exposure and AKI was also performed using the same mixed modeling and controlling for the same significant confounders. All *p* values < 0.05 were considered statistically significant.

All analyses were performed using SAS© (version 9.4; SAS institute, Cary, NC, USA). All results are reported in accordance with the STROBE guidelines [20].

## Results

### Subject selection

There were 761 VLBW infants born at MUSC or admitted to MUSC NICU within 48 h of life between 1/1/2018 and 12/31/2020. We excluded 194 patients for the following reasons: NICU admission after 48 h (*n* = 163), death within 48 h (*n* = 19), transitioned to palliative care within 48 h (*n* = 3), renal/urinary tract anomalies (*n* = 5), congenital urinary tract anomalies (*n* = 3), <2 measured serum creatinine (*n* = 1). After exclusion, 567 patients were included in our analysis.

### Postnatal steroid exposure

Out of 567 subjects included, 97 (17.1%) received PNS. The most common PNS received was hydrocortisone (68/97, 70%) followed by dexamethasone (30/97, 31%). Subjects treated with PNS had significantly lower GA, BW, and Apgar scores at 1 and 5 min, had significantly longer LOS, and were significantly more likely to die prior to discharge (Table 1). Notably, AI was diagnosed more frequently in those with PNS exposure than in those without PNS exposure (Table 1).

**Table 1:** Baseline demographics and characteristics of infants with post-natal steroid exposure versus no post-natal steroid exposure.

Baseline demographics and characteristics		Overall (*n*=567)	PNS Exposure* (*n*=97, 17.1%)	No PNS Exposure* (*n*=470, 82.9%)	*p* value°
GA (weeks)		28.4 ± 2.8	25.9 ± 2.4	28.9 ± 2.6	**<0.001**
Birthweight (grams)		1071 ± 291	813 ± 244	1125 ± 271	**<0.001**
Apgar	1 min	5 [3, 7]	3 [1, 5]	5 [3, 7]	**<0.001**
	5 min	8 [6, 8]	6 [5, 7]	8 [7, 9]	**<0.001**
Size for GA	Small	171 (30)	23 (24)	148 (32)	0.126
	Average	381 (67)	73(75)	308 (66)	0.067
	Large	14 (2.5)	1 (1)	13 (2.8)	0.315
Male		275 (49)	54 (56)	221 (47)	0.121
Race	Caucasian	172 (30)	26 (27)	146 (31)	0.406
	Black/AA	322 (57)	56 (58)	266 (57)	0.837
	Hispanic	30 (5.3)	5 (5.2)	25 (5.3)	0.948
	Other	39 (6.9)	8 (8.3)	31 (6.6)	0.559
Died prior to discharge		37 (6.5)	17 (18)	20 (4.3)	**<0.001**
Length of Stay**		60 [35, 98]	114 [93, 139]	51 [31, 83]	**<0.001**
AKI in DC diagnoses		101 (18)	27 (28)	74 (16)	**0.005**
Adrenal insufficiency		58 (10)	42 (43)	16 (3.4)	**<0.001**

Categorical variables are presented as n (%); Continuous variables as mean ± standard deviation or median [Q1, Q3]. DC: Discharge. AA: African American. °*p* values from chi-square test, Student’s *t* test, or Wilcoxon Rank Sum test. *Excludes antenatal steroid exposure. Includes post-natal steroid exposure prior to first episode of AKI. **Length of stay only includes 530/567 patients as the remaining were still admitted at time of data collection.

### Acute kidney injury

The incidence of AKI in the cohort was 22.9% (*N* = 130) though only 101 (78%) of these neonates had the diagnosis of AKI noted on their discharge summaries. The demographics describing those that did and did not develop AKI are presented in Supplementary Table 2. Those who developed AKI had significantly lower GA, BW, and Apgar scores at 1 and 5 min. Those with AKI had significantly longer LOS and were significantly more likely to die prior to discharge than those without.

### Association between postnatal steroid exposure and acute kidney injury

In our cohort, 35 infants received PNS before AKI developed and the median time between initiation of steroid exposure and diagnosis of AKI was 12 days [6, 21]. In bivariable analysis, PNS exposure was found to be associated with increased risk of AKI (RR 1.86, 95% CI 1.35–2.55; *p* < 0.01) (Supplementary Table 3). On multivariable analysis, PNS exposure was associated with a 1.72-fold increased adjusted relative risk of developing AKI in our cohort (aRR 1.72 95% CI 1.09–2.72, *p* = 0.02). Supplementary Table 3 presents the bivariable and multivariable analysis evaluating factors associated with the development of AKI. Notably, after inclusion in the multivariate model, hypotension, PDA, sepsis, and mechanical ventilation remained highly significant predictors of AKI.

### Sub-analysis: association between hydrocortisone exposure and acute kidney injury

In our cohort, 68 (11.9%) neonates received hydrocortisone. In a secondary sub-analysis, HC appeared to have a protective effect against AKI with the incidence of AKI in neonates treated with HC being 23%, compared to 77% in those without HC exposure (*p* < 0.01, Table 2). However, after controlling for significant confounders and baseline differences, HC exposure was no longer associated with AKI (aRR 1.51, 95% CI 0.91–2.52, Table 3).

**Table 2:** Bivariable association between acute kidney injury and hydrocortisone exposure.

Post-natal hydrocortisone	AKI (*n*=130)	No AKI (*n*=437)	*p* value°
Hydrocortisone Exposure* (*n*=68)	30 (23.1)	38 (8.7)	**<0.001**
No Hydrocortisone Exposure* (*n*=499)	100 (76.9)	399 (91.3)	

*Includes hydrocortisone exposure prior to first episode of AKI. °*P* value from Chi-square test.

**Table 3:** Associations between hydrocortisone exposure and acute kidney injury using multivariable generalized linear mixed modeling.

Characteristic		Unadjusted relative risk (RR)	95% confidence interval	*p* value°	Adjusted relative risk (aRR)*	95% confidence interval	*p* value°
Post-natal hydrocortisone exposure		2.20	(1.60, 3.03)	**<0.001**	1.51	(0.91, 2.52)	0.114
Small for Gestational Age		0.72	(0.69, 0.76)	**<0.001**	0.96	(0.84, 1.09)	0.509
Birth weight (grams)		0.99	(0.997, 0.998)	**<0.001**	0.99	(0.99, 1.00)	0.143
Apgar	1 min	0.85	(0.80, 0.91)	**<0.001**	0.99	(0.90, 1.09)	0.886
	5 min	0.85	(0.80, 0.90)	**<0.001**	1.03	(0.91, 1.18)	0.607
Adrenal insufficiency		3.11	(2.34, 4.12)	**<0.001**	1.07	(0.62, 1.82)	0.811
Caffeine		7.00	(2.64, 18.53)	**<0.001**	1.12	(0.38, 3.27)	0.835
Hypotension		4.21	(3.17, 5.59)	**<0.001**	1.53	(1.08, 2.17)	**0.018**
Patient ductus arteriosus		4.81	(3.30, 7.03)	**<0.001**	1.66	(1.12, 2.46)	**0.011**
Sepsis		3.97	(3.00, 5.24)	**<0.001**	1.50	(1.07, 2.10)	**0.018**
Mechanical ventilation		11.52	(5.74, 23.13)	**<0.001**	3.79	(1.74, 8.29)	**<0.001**
Necrotizing enterocolitis		2.33	(1.68, 3.23)	**<0.001**	1.33	(0.80, 2.21)	0.277
Nephrotoxic medication exposure		7.10	(3.19, 15.79)	**<0.001**	1.73	(0.74, 4.07)	0.209

*Controlled for small for gestational age, birthweight, Apgar scores, death prior to discharge, hypotension, patent ductus arteriosus, sepsis, and mechanical ventilation. °*P* values from bivariate and multivariate generalized mixed modeling.

## Discussion

This study is the largest study to date evaluating associations between PNS and AKI in VLBW neonates. In the current study we show that PNS exposure and AKI occur commonly in this population. Furthermore, we show that in our cohort, PNS are associated with an increased incidence of AKI. However, when evaluated individually, hydrocortisone initially was associated with decreased AKI, but this association was no longer present after multivariable analysis. These results highlight the need for further study evaluating the impact of PNS on the incidence of AKI and associated outcomes.

AKI is a common occurrence in the NICU and associated with increased morbidity and mortality in critically ill neonates. AKI prevalence in infants born extremely prematurely is as high as 48% [1]. These same extremely premature neonates who are at high risk for AKI frequently are exposed to PNS, which could impact kidney health. In the current study we sought to address this knowledge gap by examining associations between PNS and neonatal AKI using a large cohort of VLBW neonates cared for in a NICU with protocolized AKI care. Our findings add to the findings of prior studies of neonatal AKI [22] and corroborate those of other investigators. We found neonatal AKI is significantly more common in infants with lower GA, BW, and APGAR scores, those with AKI had significantly longer LOS and those with AKI were significantly more likely to die prior to discharge. Independent risk factors for AKI in our cohort included hypotension, PDA, sepsis, and mechanical ventilation. Similarly, we found subjects treated with PNS had significantly lower GA, BW, and Apgar scores at 1 and 5 min, had significantly longer LOS, and were significantly more likely to die prior to discharge. Our novel findings include a 1.72-fold increased adjusted relative risk of developing AKI in those infants exposed to PNS, which was contrary to our hypothesis. We also found that infants with AKI were significantly more likely to have been diagnosed with AI than those without AKI, though we note that AI could certainly have gone undiagnosed in our cohort, particularly in those to whom PNS were prescribed. Lastly, in performing a sub-analysis on HC exposure and associations with AKI, we detected a protective association between HC exposure and AKI; however, after controlling for significant confounders, this association was no longer present.

Our hypothesis that PNS exposure is associated with decreased incidence of AKI was based on both the findings of Baserga et al. and Shimokaze et al., as well as the underlying physiology of relative or transient AI [11, 12]. Shimokaze et al. investigated late-onset glucocorticoid-responsive circulatory collapse (LCC) in VLBW infants, an event characterized by sudden onset hypotension and/or oliguria after 7 days of life, which is resistant to volume expanders and inotropes but responds rapidly to intravenous glucocorticoids. They found that 14 of 41 infants (34%) in their cohort developed LCC with AKI (primarily documented with decreased UOP) and all of these infants had resolution with the initiation of HC. Baserga et al. looked similarly at VLBW infants during this time frame that developed AKI, however without the circulatory collapse. They found that 27 of 230 (11.7%) infants developed AKI without clear underlying etiology, all of whose AKI resolved with the initiation of HC. Both groups concluded that relative AI in these infants could be the cause of their AKI. Further articles describe LCC and investigate the pathophysiology, hypothesizing again its relation to relative adrenal insufficiency in this population [13, 14]. Fofi et al. described the importance of the renin-angiotensin-aldosterone system (RAAS) in renal homeostasis and the role that RAAS disruption may play in the development of AKI in adult patients. In patients with AI, a combination of low aldosterone levels causing hypovolemia and low cortisol levels leading to decreased tone of the efferent renal arterioles with subsequent reduced perfusion pressure could together lead to AKI [23]. Although not reported, logic suggests that this mechanism would apply to preterm infants as well. Extremely preterm neonates likely also have an immature hypothalamic-pituitary-adrenal (HPA) axis. The HPA axis is functional in the fetus by 20 weeks of gestation but does not fully mature until after 30 weeks of gestation. The administration of exogenous glucocorticoids suppresses cortisol production and secretion of cortisol through negative feedback on this immature HPA axis, potentially leading to suppression of this HPA axis and subsequent AI [24].

Our findings did not support our hypothesis. Rather than being protective against AKI, we found that PNS exposure is associated with increased risk of AKI development. We suspect the detected association between PNS exposure and AKI may be partially confounded by indication and the inclusion of multiple types of PNS. Despite our efforts to control for degree of illness and AKI confounders in our analysis, it is possible that PNS exposure is a marker of clinical severity of illness in these patients and subsequently their increased risk for AKI. However, the median length of time between steroid initiation and AKI diagnosis of 12 days indicates a prolonged separation between the presumed acute illness necessitating start of steroids and an AKI diagnosis, potentially making this confounding less likely. Given the potential links between the RAAS pathway, the immature HPA axis and AKI in the premature infant, as discussed above, it is possible that in some cases, neonates may experience underlying undiagnosed AI; when these infants receive PNS, it may serve a renal protective effect while the infant is exposed, and AKI occurs in these infants when the steroids are discontinued leading us to detect a misleading association between PNS exposure and AKI. The prolonged median 12-day period between steroid initiation and AKI diagnosis may additionally lend merit to this hypothesis, as possibly these infants had begun a steroid wean or even had their steroids discontinued during this timeframe. AI is difficult to diagnose in the premature neonate, as cortisol levels do not appear to correlate with disease severity and most infants are diagnosed based on clinical symptoms (hyponatremia, hyperkalemia, hypotension, oliguria) [25]. This may lead to an underdiagnosis of AI which may cause an under recognition of infants at risk for development of AKI post-PNS exposure. Though we found a strong association between AI and risk of AKI in bivariable analysis, this association was no longer present in adjusted analysis potentially due to underdiagnosis of AI.

In a sub-analysis of associations between solely HC exposure and AKI, a protective association was found between HC exposure and AKI. However, after controlling for significant confounders, HC exposure alone was no longer associated with AKI. There are several potential reasons why we found an association with all PNS exposure, but not HC alone. First, dexamethasone, the most common steroid in the PNS exposure group besides hydrocortisone, is 40-50 times stronger than HC and longer-acting [21], potentially creating more suppression on the HPA axis and leading to an iatrogenic AI in these infants. Second, as only 68 of 567 infants received HC, there is a potential the sample size was too small to detect an association, and with a larger sample size we may have seen the protective association between HC and AKI stand even in an adjusted analysis.

Limitations of our study include the observational and retrospective design. Intervals between serum creatinine evaluations were at provider discretion and not protocolized. Similarly, PNS prescription was at the discretion of the provider. In the absence of a more detailed understanding of the timing, indication for, and type of steroid administered, conclusions based on the detected association between steroid exposure and AKI are limited. However, given the scarcity of available literature and studies examining this association, these findings bear consideration. Though we determined that several confounding factors were associated with risk of AKI, additional confounders may exist that were not examined. Additionally, AI diagnosis was also at the discretion of the provider and can be difficult to diagnosis.

In conclusion, the current study shows that PNS exposure was associated with a 1.72-fold increased adjusted relative risk of developing AKI in our cohort. HC exposure alone was not associated with increased risk of AKI. These findings should be interpreted with caution and warrant further detailed study as the relationship between AI, PNS, and indication for PNS treatment is extraordinarily complex. In future work, it will be important to investigate both timing, type, and medical indication for PNS exposure when examining associations with AKI, as well as including timing of AKI whether it be closely following delivery (as in late onset glucocorticoid-responsive circulatory collapse) or later in the infant’s course. It will be important to examine PNS dosing and duration of treatment, as well as temporal association with AKI diagnosis including if the patient was still on PNS at time of AKI and/or length of time from discontinuation to diagnosis of AKI. It may also be prudent to examine re-initiation of PNS and subsequent resolution of AKI. It will also be important to utilize consistent criteria to diagnose AI to evaluate associations more effectively.

## Supplementary information

Neonatal Acute Kidney Injury Diagnostic Criteria using Modified, Neonatal Kidney Disease: Improving Global Outcomes (KDIGO) Criteria

Supplemental Table 2. Baseline Demographics and Characteristics of Infants with Acute Kidney Injury versus no Acute Kidney Injury

Supplemental Table 3. Associations between Post-natal Steroid Exposure and Acute Kidney Injury using Multivariable Generalized Linear Mixed Modeling

## Data Availability

These data are not publicly available due to IRB limitations.

## Study approval statement

This study protocol was reviewed and approved by the Medical University of South Carolina Institutional Review Board for Human Research, approval number Pro00107979. This study was exempt from ethics committee approval per the IRB review.
