# Supplementary material for: Post-natal steroid exposure in very low birthweight neonates and associations with acute kidney injury
Source: J Perinatol. 2024 May 23;44(12):1786–91. doi: 10.1038/s41372-024-02011-4 (PMC11606920; doi:10.1038/s41372-024-02011-4)
Supplement: Supplementary file 1 — Neonatal Acute Kidney Injury Diagnostic Criteria using Modified, Neonatal Kidney Disease: Improving Global Outcomes (KDIGO) Criteria [file 41372_2024_2011_MOESM1_ESM.docx]

| **AKI Stage** | **Serum Creatinine (SCr) Criteria** |
| --- | --- |
| **0** | No change in SCr *or*  SCr rise <0.3 mg/dL |
| **1** | SCr rise ≥0.3 mg/dL rise within 48 hrs *or* SCr rise ≥1.5–1.9 × baseline SCr^a^ |
| **2** | SCr rise ≥2.0–2.9 × baseline SCr^a^ |
| **3** | SCr rise ≥3 × baseline SCr^a^ *or* SCr ≥2.5 mg/dL^b^ *or* Kidney Support Therapy Utilization |

**Supplemental Table 1.** Neonatal Acute Kidney Injury Diagnostic Criteria using Modified, Neonatal Kidney Disease: Improving Global Outcomes (KDIGO) Criteria

*^a^Baseline SCr defined as lowest previous SCr value. ^b^SCr value of 2.5 mg/dL represents glomerular filtration rate of <10 mL/min/1.73m^2^. SCr, serum creatinine; mg/dL, milligrams per deciliter; hrs, hours. Adapted from Kidney Disease: Improving Global Outcomes (KDIGO) Acute Kidney Injury Workgroup. KDIGO clinical practice guideline for acute kidney injury. Kidney Int Suppl 2012;2:1–138.*
