# Supplementary material for: Post-natal steroid exposure in very low birthweight neonates and associations with acute kidney injury
Source: J Perinatol. 2024 May 23;44(12):1786–91. doi: 10.1038/s41372-024-02011-4 (PMC11606920; doi:10.1038/s41372-024-02011-4)
Supplement: Supplementary file 2 — Supplemental Table 2. Baseline Demographics and Characteristics of Infants with Acute Kidney Injury versus no Acute Kidney Injury [file 41372_2024_2011_MOESM2_ESM.docx]

| **Baseline Demographics and Characteristics** | | **Overall**  **(n=567)** | **AKI**  **(n=130, 22.9%)** | **No AKI**  **(n=437, 77.1%)** | **p-value*** |
| --- | --- | --- | --- | --- | --- |
| **GA (weeks)** | | 28.4 ± 2.8 | 26.1 ± 2.4 | 29.1 ± 2.5 | **<.001** |
| **Birthweight (grams)** | | 1071 ± 291 | 835 ± 246 | 1142 ± 265 | **<.001** |
| **Apgar** | 1 minute | 5 [3, 7] | 4 [2, 6] | 5 [3, 7] | **<.001** |
|  | 5 minutes | 8 [6, 8] | 7 [6, 8] | 8 [7, 9] | **<.001** |
| **Size for GA** | Small | 171 (30) | 28 (22) | 143 (33) | **0.014** |
|  | Average | 381 (67) | 98 (75) | 283 (65) | **0.025** |
|  | Large | 14 (2.5) | 4 (3.1) | 10 (2.3) | 0.614 |
| **Male** | | 275 (49) | 72 (55) | 203 (47) | 0.074 |
| **Race** | Caucasian | 172 (30) | 36 (28) | 136 (31) | 0.455 |
|  | Black/AA | 322 (57) | 77 (59) | 245 (56) | 0.522 |
|  | Hispanic | 30 (5.3) | 11 (8.5) | 19 (4.4) | 0.066 |
|  | Other | 39 (6.9) | 6 (4.6) | 33 (7.6) | 0.246 |
| **Died prior to discharge** | | 37 (6.5) | 24 (19) | 13 (3.0) | **<.001** |
| **Length of Stay**** | | 60 [35, 98] | 103 [83, 140] | 51 [30, 83] | **<.001** |
| **AKI in DC diagnoses** | | 101 (18) | 79 (61) | 22 (5.0) | **<.001** |
| **Adrenal insufficiency** | | 58 (10) | 34 (26) | 24 (5.5) | **<.001** |

**Supplemental Table 2.** Baseline Demographics and Characteristics of Infants with Acute Kidney Injury versus no Acute Kidney Injury

*Legend: Categorical variables are presented as n (%); Continuous mean ± standard deviation or median [Q1, Q3]. DC: Discharge. AA: African American. *p-values from chi-square test, Student’s t-test, or Wilcoxon Rank Sum test. **Length of stay only includes 530/567 patients as the remaining were still admitted at time of data collection.*
