# Supplementary material for: Post-natal steroid exposure in very low birthweight neonates and associations with acute kidney injury
Source: J Perinatol. 2024 May 23;44(12):1786–91. doi: 10.1038/s41372-024-02011-4 (PMC11606920; doi:10.1038/s41372-024-02011-4)
Supplement: Supplementary file 3 — Supplemental Table 3. Associations between Post-natal Steroid Exposure and Acute Kidney Injury using Multivariable Generalized Linear Mixed Modeling [file 41372_2024_2011_MOESM3_ESM.docx]

| **Characteristic** | | **Unadjusted Bivariate Comparison** | | | **Adjusted Multivariate Comparison** | | |
| --- | --- | --- | --- | --- | --- | --- | --- |
|  |  | Relative Risk (RR) | RR 95% Confidence Interval | p-value**°** | Adjusted Relative Risk (aRR)* | aRR 95% Confidence Interval | p-value**°** |
| **Post-natal steroid use**** | | 1.86 | (1.35, 2.55) | **<.001** | 1.72 | (1.09, 2.72) | **0.021** |
| **Gestational age (weeks)** | | 0.72 | (0.69, 0.76) | **<.001** | 0.91 | (0.83, 1.00) | 0.058 |
| **Small for Gestational Age** | | 0.63 | (0.43, 0.93) | **0.018** | 1.01 | (0.71, 1.43) | 0.962 |
| **Apgar** | 1 minute | 0.85 | (0.80, 0.91) | **<.001** | 0.99 | (0.89, 1.09) | 0.777 |
|  | 5 minutes | 0.85 | (0.80, 0.90) | **<.001** | 1.01 | (0.90, 1.15) | 0.814 |
| **Adrenal insufficiency** | | 3.11 | (2.34, 4.12) | **<.001** | 1.10 | (0.67, 1.81) | 0.713 |
| **Caffeine** | | 6.99 | (2.64, 18.53) | **<.001** | 1.15 | (0.40, 3.32) | 0.791 |
| **Hypotension** | | 4.21 | (3.17, 5.59) | **<.001** | 1.64 | (1.14, 2.36) | **0.001** |
| **Patent ductus arteriosus** | | 4.81 | (3.30, 7.03) | **<.001** | 1.76 | (1.18, 2.64) | **0.006** |
| **Sepsis** | | 3.97 | (3.00, 5.24) | **<.001** | 1.51 | (1.06, 2.15) | **0.023** |
| **Mechanical ventilation** | | 11.52 | (5.74, 23.13) | **<.001** | 4.05 | (1.87, 8.78) | **<.001** |
| **Necrotizing enterocolitis** | | 2.33 | (1.68, 3.23) | **<.001** | 1.31 | (0.79, 2.18) | 0.294 |
| **Nephrotoxic medication exposure** | | 7.10 | (3.19, 15.79) | **<.001** | 1.71 | (0.74, 3.98) | 0.211 |

**Supplemental Table 3.** Associations between Post-natal Steroid Exposure and Acute Kidney Injury using Multivariable Generalized Linear Mixed Modeling

*Legend: *Controlled for small for gestational age, birthweight, one-minute Apgar score, death prior to discharge, hypotension, patent ductus arteriosus, sepsis, and mechanical ventilation. **Includes post-natal steroid exposure prior to first episode of AKI. °P-values from bivariate and multivariate generalized mixed modeling.*
